# Epidemiological characteristics of a protracted and widespread measles outbreak in the Democratic Republic of Congo, 2018 - 2020

**DOI:** 10.11604/pamj.2022.42.282.34410

**Published:** 2022-08-15

**Authors:** Vincent Dossou Sodjinou, Marcellin Nimpa Mengouo, Alfred Douba, Patricia Tanifum, Moise Desire Yapi, Kanyiga Robert Kuzanwa, John Samuel Otomba, Balcha Masresha

**Affiliations:** 1 World Health Organization Regional Office for Africa, Brazzaville, Congo,; 2 World Health Organization Country Office for Democratic Republic of Congo, Kinshasa, the Democratic Republic of the Congo,; 3 Public Health Department, Felix Houphouet Boigny University, Abidjan-Cocody, Cote d'Ivoire,; 4 World Health Organization Intercountry Team for Central Africa, Libreville, Gabon

**Keywords:** Measles, outbreak, elimination, vaccination, Democratic Republic of Congo, epidemiological trends

## Abstract

**Introduction:**

measles is a highly contagious viral disease. Since 2011, the Democratic Republic of Congo (DR Congo) has had the first dose measles vaccination coverage of less than 80% according to the World Health Organization - United Nations International Children's Emergency Fund (WHO-UNICEF) coverage estimates, and measles mass vaccination coverage of less than the required coverage level of 95% by survey. Starting in August 2018, the country experienced an increase in measles case reports which continued through to early 2020. Epidemiological aspects of the outbreak are described in this article.

**Methods:**

we analysed aggregate weekly passive surveillance data from the DR Congo for the years 2018 - 2020 to understand the trends of occurrence of suspected measles cases. We also analysed the measles case-based surveillance database to understand the epidemiological characteristics of confirmed cases of measles and rubella during the same period of time.

**Results:**

a total of 458,156 suspected measles cases and 8,127 deaths were reported between 1^st^ January 2018 and 31^st^ December 2020, with the majority of cases and deaths reported in 2019. Only 2.9% of these cases were reported through the case-based surveillance system, with 31,639 cases being confirmed as measles by the laboratory, by epidemiological linkage and on clinical compatibility. Children less than 5 years of age were most affected with a cumulative incidence of 960 cases per 1,000,000 inhabitants. Only 41% of the confirmed cases were vaccinated. Maindombe and Tshopo provinces had the highest cumulative incidence levels. There was a distinct geographic progression of the outbreak between provinces during the course of the three years. A total of 1760 lab confirmed rubella cases were confirmed in various provinces among the cases investigated with blood specimens, 93% of whom were less than 15 years of age.

**Conclusion:**

the recent gaps in vaccination coverage, the age pattern of confirmed cases and the lack of vaccination history in the majority of cases is suggestive of failure to vaccinate as the likely cause of this large and protracted outbreak. Efforts to improve vaccination coverage and the measles surveillance system are needed in order to prevent the occurrence of future outbreaks and to avert measles-related deaths.

## Introduction

Measles is an acute and highly contagious viral disease with a basic reproductive number (the average number of secondary cases produced by a primary case in a completely susceptible population) of 12-18. Children are mostly affected. Complications of measles include encephalitis, pneumonia, ear infections, persistent diarrhea, upper airway obstruction, mouth ulcers, blindness and death [1-4].

Measles outbreaks are among the commonly reported disease outbreaks in the African Region of the World Health Organization (WHO) [5,6]. Before the introduction of the measles vaccine in 1963, major epidemics occurred approximately every 2 to 3 years and it is estimated that 30 million cases of measles and more than 2 million deaths occurred globally each year [1]. In 2012, the global vaccine action plan approved by the World Health Assembly set a goal for measles and rubella elimination in at least 5 of the 6 WHO regions by 2020 [7,8].

In 2011, the World Health Organization (WHO) African Region adopted a strategy and a resolution for measles elimination in the region by 2020. The targets adopted for 2020 are: attaining measles incidence of less than 1 case per million population; maintaining 95% measles immunization coverage at national level and in all districts; attaining 95% coverage in all scheduled measles supplementary immunization activities (SIAs) and in response to outbreaks; and maintaining the targets for the two main surveillance performance indicators [9]. These indicators include an annual non-measles febrile rash illness rate of at least 2 per 100,000 population and 80% or more districts reporting at least 1 suspected case of measles with a blood specimen every year [10]. Since the launch of measles control strategies, there has been some progress towards measles elimination in the African Region. The number of estimated measles cases decreased from 10,727,500 in 2000 to 4,548,000 in 2019, and estimated measles deaths dropped from 346,400 in 2000 to 147,900 in 2019. This represents an estimated mortality reduction of 57% over that time period [11].

In 2016, an evaluation of the progress of African countries towards measles elimination by 2020 showed that the Democratic Republic of Congo (DR Congo) was among the countries significantly off-track for achieving the elimination goal [12]. In the DR Congo, between 2012 and 2019, measles vaccine administrative coverage varied between 88% and 92%, while the WHO-UNICEF estimates of coverage with the first dose of measles vaccine (MCV1) ranged between 57% and 72% [13]. As of the end of 2021, the second dose of measles vaccine is not yet included in the national routine immunization schedule in the DR Congo. Periodic supplementary measles immunization activities (SIAs) were conducted repeatedly in DR Congo, but did not reach the minimum coverage target of 95% in some areas. In December 2015, a post campaign coverage survey in the provinces of Katanga, Kasaï Oriental and Nord Kivu showed a coverage of 91% [14]. Another post-campaign coverage survey conducted in 2016 in Maniema Province indicated measles SIAs coverage was 82% in the urban areas and 91% in the rural areas [15]. A prolonged humanitarian crisis in the eastern part of the country weakened the health system with millions of people lacking access to health services including immunization services [16-19]. It is also documented that malnutrition affects more than 50% of children under 5 in the DR Congo [20].

The aggregate disease reporting system in DR Congo continues to document measles as a major public health problem, even though the quality of the measles case-based surveillance system remains weak [21]. Prolonged and multiple measles outbreaks have affected different parts of the country in the course of the years 2011 - 2015 [22,23]. At the end of the year 2018, outbreaks of measles were reported in the eastern parts of the country, and following a spread into other provinces, it was officially declared by the government on 10^th^ June 2019 [24]. We describe the epidemiological aspects of this outbreak in this article.

## Methods

**Study design:** a descriptive study of the occurrence of measles in DR Congo using surveillance data from 2018 - 2020.

**Study setting:** the Democratic Republic of Congo has a surface area of 2,345,409 km^2^. The health infrastructure consists of 26 provincial health divisions and 516 health zones at peripheral level [25].

**Disease surveillance system:** the national epidemiological surveillance system in DR Congo is run by the department of epidemiological surveillance in the ministry of health, and is responsible for the weekly aggregate notification of diseases and the control of epidemic prone diseases. The aggregate reporting of diseases is a passive reporting system that provides information on the weekly trends of occurrence of suspected cases of notifiable diseases. The aggregate surveillance database does not contain detailed epidemiological information on reported cases except for the number of cases by week of reporting, the reporting district, and the age categorization of less than 5 years of age, or 5 years and above.

The case-based measles surveillance system with laboratory confirmation has been operational in all provinces of the country since 2006. This measles surveillance is part of the Integrated Disease Surveillance and Response (IDSR) system, which uses two strategies, namely active surveillance of cases from notification sites and in the community, as well as passive surveillance.

**Blood sample collection and laboratory testing:** within the measles case-based surveillance system, as soon as 3 samples of suspected cases are confirmed positive for measles immunoglobulin M (IgM) by the laboratory, all other cases in the same area with dates of onset of rash within 30 days are confirmed by epidemiological linkage and captured in district line-lists.

Serum samples were collected from suspected measles cases within 30 days of rash onset. Specimens are shipped to one of the two national measles serological laboratories, in Kinshasa or in Lubumbashi, for confirmation. Laboratory confirmation of measles was made by detection of measles immunoglobulin M (IgM) antibody using a standard commercial enzyme immunoassay indirect kit. All specimens negative for measles IgM are systematically tested for the presence of rubella IgM antibodies [26].

**Case definitions:** a suspected measles case was defined as: i) any person with generalized maculo-papular rash and fever plus one of the following: cough or coryza (runny nose) or conjunctivitis (red eyes); ii) any person in whom a clinician suspects measles. A laboratory confirmed measles was defined as a suspected measles case that is investigated, including the collection of blood specimen, has serological confirmation of recent measles virus infection (measles IgM positive) and had not received measles vaccination in the 30 days preceding the specimen collection. Measles confirmed by epidemiological linkage was defined as a suspected measles case that has not had a specimen taken for serologic confirmation and is linked (in place, person and time) to lab confirmed cases; i.e. living in the same or in an adjacent district with a lab confirmed case where there is a likelihood of transmission; onset of rash of the two cases being within 30 days of each other. A confirmed outbreak of measles was defined as 3 or more measles IgM positive (laboratory confirmed) cases in a health facility or district in one month [10].

**Variables:** variables in the case-based surveillance reporting forms, and in the line lists included the name of health district, year, suspected disease, epidemiological week, patient´s name, health facility´s name, residence, sex, age, date of rash onset, health facility visit date, symptoms, immunization status, lab test, and outcome. The measles vaccination status of the cases was confirmed by history from the parents or guardians accompanying the child to the health facility. During the 2018-2020 measles outbreak, data was collected using line lists, through the aggregate weekly surveillance reporting database, and through the case-based surveillance system. Line lists were available in health facilities.

**Statistical methods:** we analyzed the aggregate surveillance reports database (commonly referred to as the IDSR data) and the case-based measles surveillance databases kept at the national level. We characterized the epidemiological characteristics of measles cases reported from 1^st^ January 2018 to 31^st^ December 2020. Data were analyzed using EPI-INFO version 3.5.4 for Windows, and MS Excel. Frequency statistics were used to describe the epidemiological characteristics in different population subgroups. Case fatality ratios and incidence rates were generated.

## Results

**Sociodemographic characteristics of suspected measles cases reported in the aggregate surveillance database:** in the period from 1^st^ January 2018 to 31^st^ December 2020, a total of 458,156 measles suspected cases and 8,127 measles deaths were reported according to the aggregate surveillance reports (the IDSR database). The cumulative total number of cases reported during this period ranged from 2,458 cases in Kwango Province to 50,969 cases in Tshopo- the most affected province. Forty six percent of the total cases were reported from six provinces (Tshopo, Kasai, Maindombe, Haut Lomami, Kwilu and South Kivu). The cumulative incidence of reported measles was 4,070 per million population, with the highest cumulative incidence over the three years period reported in Maindombe and Tshopo provinces (15,295 and 13,805 cases per million population respectively).

**Figure 1:**
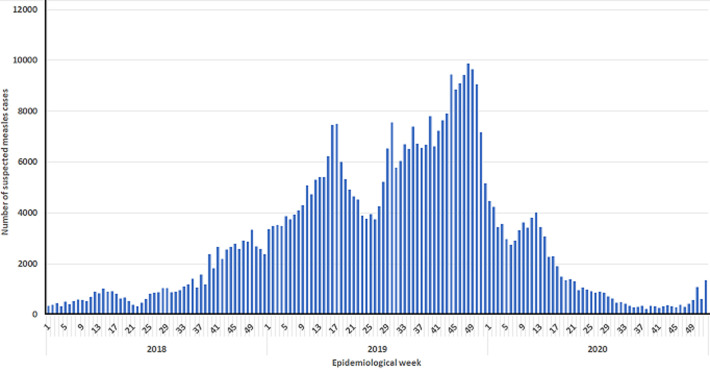
epidemiological curve of suspected measles cases and deaths, DR Congo, January 2018 - December 2020

During the three years period, a total of 8,127 measles deaths were reported with a case fatality ratio of 1.8% for the whole country. Seven provinces (Kasai, Tshopo, Maindombe, Kwilu, Kasai Central, Haut-Lomami and Sankuru) reported nearly two thirds (63%) of the measles deaths. Sankuru and Kasai Central provinces had the highest case fatality ratio of 4.9% and 3.9% respectively (Table 1). The number of reported cases and deaths was highest in 2019, with 312,409 cases (68.2% of the cases) and 6062 deaths (74.6% of the deaths). The weekly occurrence of suspected measles cases shows two major peaks in 2019 around week 17 and week 48 (Figure 1).

**Table 1:** suspected measles cases and deaths per province, 2018 - 2020, DR Congo; aggregate surveillance data

Health zone	Population 2020	2018		2019		2020		Cumulative cases 2018 - 2020			Cumulative incidence per 1,000,000 population
		Cases	Deaths	Cases	Deaths	Cases	Deaths	Cases	Deaths	Case fatality ratio	
Bas-Uele	1,408,165	38	NA	5,483	168	5,175	93	10,696	261	2.4%	7,596
Equateur	2,829,783	186	2	12,609	312	5,611	108	18,406	422	2.3%	6,504
Haut-Katanga	6,668,993	9,598	147	2,325	15	542	0	12,465	162	1.3%	1,869
Haut-Lomami	4,273,260	11,143	152	19,429	396	1,693	3	32,265	551	1.7%	7,550
Haut-Uele	2,235,374	575	5	8,482	107	1,900	10	10,957	122	1.1%	4,902
Ituri	6,409,236	4,319	46	11,043	66	1,221	2	16,583	114	0.7%	2,587
Kasai	5,374,391	242	7	36,614	1,023	3,038	57	39,894	1,087	2.7%	7,423
Kasai Central	5,926,889	166	4	13,525	561	1,163	13	14,854	578	3.9%	2,506
Kasai Oriental	5,337,463	4,786	50	11,899	125	1,534	4	18,219	179	1.0%	3,413
Kinshasa	9,994,530	222	1	8,277	137	273	0	8,772	138	1.6%	878
Kongo Central	4,246,506	199	NA	10,190	73	4,720	20	15,109	93	0.6%	3,558
Kwango	2,690,400	104	NA	2,028	32	326	2	2,458	34	1.4%	914
Kwilu	5,680,874	60	NA	25,266	586	3,524	53	28,850	639	2.2%	5,078
Lomami	4,254,293	476	11	8,840	74	237	1	9,553	86	0.9%	2,245
Lualaba	2,878,904	6,090	73	9,717	54	200	9	16,007	136	0.8%	5,560
Maindombe	2,159,606	51	1	25,702	710	7,279	113	33,032	824	2.5%	15,295
Maniema	2,938,101	3,466	28	2,625	31	1,114	11	7,205	70	1.0%	2,452
Mongala	2,952,853	239	2	9,768	96	6,336	44	16,343	142	0.9%	5,535
Nord-Kivu	9,539,901	486	3	7,488	19	5,009	30	12,983	52	0.4%	1,361
Nord-Ubangi	1,693,624	60	2	387	7	6,771	103	7,218	112	1.6%	4,262
Sankuru	2,553,626	1,353	77	3,115	157	6,744	315	11,212	549	4.9%	4,391
Sud Ubangi	7,916,500	108	1	3,537	44	7,530	37	11,175	82	0.7%	1,412
Sud-Kivu	3,396,077	3,882	19	19,723	160	2,346	35	25,951	214	0.8%	7,641
Tanganyika	3,266,819	2,236	22	9,740	164	1,500	50	13,476	236	1.8%	4,125
Tshopo	3,692,007	15,127	217	33,529	628	2,313	21	50,969	866	1.7%	13,805
Tshuapa	2,259,852	85	2	11,068	317	2,351	59	13,504	378	2.8%	5,976
Grand total	112,578,026	65,297	872	312,409	6,062	80,450	1193	458,156	8,127	1.8%	4,070

NA: not applicable

**Figure 2:**
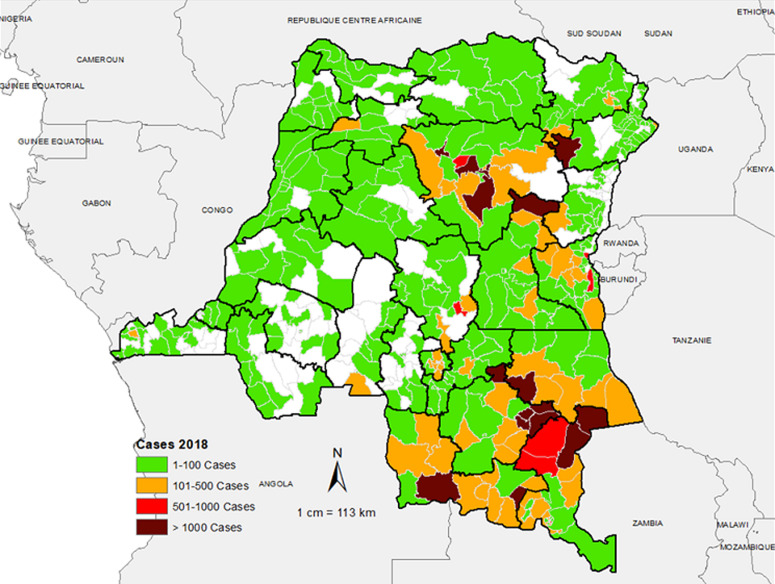
number of suspected measles cases reported by health zone, 2018, DR Congo

**Figure 3:**
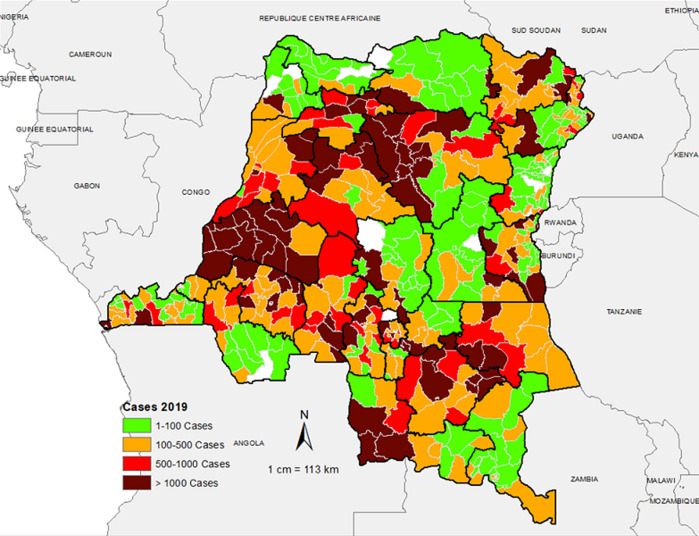
number of suspected measles cases reported by health zone, 2019, DR Congo

**Figure 4:**
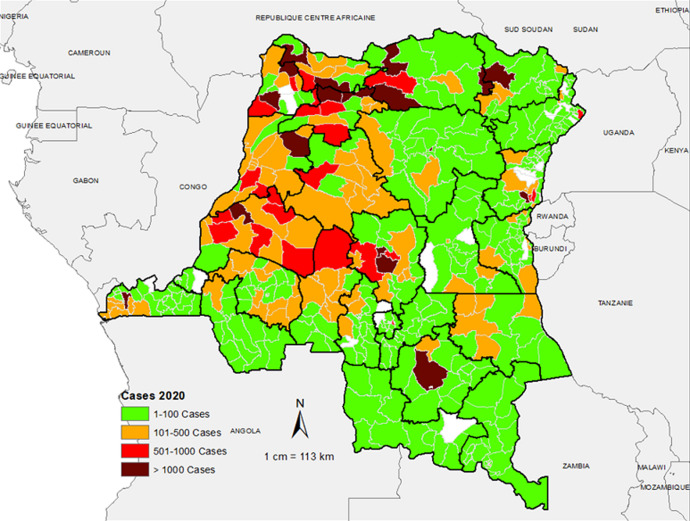
number of suspected measles cases reported by health zone, 2020, DR Congo

**Spatio-temporal distribution of suspected measles cases reported in the aggregate surveillance database:** the distribution of suspected measles from the aggregate surveillance database indicates that in 2018, the outbreak was noted in provinces in the eastern part of the country. Over the course of 2019, the outbreak spread wider with a large number of suspected cases reported from the majority of the provinces in the country. In 2020, the large case load was mostly across western part of the country, especially in the first half of the year (Figure 2, Figure 3, Figure 4). The outbreak eventually affected all the provinces in the county, with 253 health zones (49% of the 519 health zones in the country) reporting laboratory confirmed measles outbreaks.

**Epidemiological characteristics of cases reported in the case-based surveillance data:** of the total suspected cases reported from 2018 to 2020, laboratory specimens were collected from only 13,414 suspected measles cases (2.9% of the total suspected cases reported in the aggregate reporting system) over the course of the three years. Of these only 5,487 (40.9%) cases were laboratory confirmed to be measles. A total of 31,639 cases were confirmed to be measles by laboratory, by epidemiological linkage and by clinical compatibility. Of these, children under the age of five years accounted for 67%, with a cumulative incidence rate of 960 per million population over the period of three years. Children aged 5 to 9 years were the second largest group with 19% of the total and a cumulative incidence rate of 334 per million. Only 40.7% of the confirmed measles cases had a history of prior measles vaccination. In the same time period, 1760 rubella lab confirmed cases were detected, 45% of which were less than 5 years of age and 36% were aged 5 - 9 years. Only 7.4% of the confirmed rubella cases occurred among persons aged more than 15 years (Table 2). While rubella viruses were detected across many provinces throughout the 3-years period, the provinces that were mostly affected were Katanga, Kinshasa and Orientale in 2018 (with 46% of the 297 cases); Equateur, Orientale, North Kivu and Bandundu in 2019 (with 56% of the 560 cases); Kasai, Kasai Orientale, Sud Ubangi and North Kivu in 2020 (with 36% of the 903 rubella confirmed cases).

**Table 2:** confirmed measles and rubella cases and incidence of measles by age group, 2018 - 2020, DR Congo

Year	Age group	Population	Total laboratory confirmed rubella cases	Total confirmed measles cases ( lab confirmed + epidemiologically linked + clinically compatible cases)	Number of measles cases with at least one dose of measles vaccination	Proportion of vaccinated among the confirmed measles cases	Incidence of confirmed measles per million population
2018	<5 years	20,262,975	125	3499	795	22.7%	172.68
	5 - 9 years	16,314,293	131	945	202	21.4%	57.92
	10 - 15 years	13,404,737	21	115	17	14.8%	8.58
	>15 years	53,930,688	10	110	12	10.9%	2.04
	Total	03,912,693	287	4669	1026	22.0%	44.93
2019	<5 years	20,932,827	260	9897	5483	55.4%	472.80
	5 - 9 years	16,853,610	206	1780	825	46.3%	105.62
	10 - 15 years	13,847,870	69	389	154	39.6%	28.09
	>15 years	55,713,525	30	374	42	11.2%	6.71
	Total	07,347,832	565	12440	6504	52.3%	115.88
2020	<5 years	21,952,715	409	7677	3822	49.8%	349.71
	5 - 9 years	17,674,750	298	3182	895	28.1%	180.03
	10 - 15 years	14,522,565	111	1563	317	20.3%	107.63
	>15 years	58,427,996	90	2108	301	14.3%	36.08
	Total	112,578,026	908	14530	5335	36.7%	129.07
Cumulative over 3 years	<5 years	21,952,715	794	21073	10100	47.9%	959.93
	5 - 9 years	17,674,750	635	5907	1922	32.5%	334.21
	10 - 15 years	14,522,565	201	2067	488	23.6%	142.33
	>15 years	58,427,996	130	2592	355	13.7%	44.36
	Total	112,578,026	1760	31639	12865	40.7%	281.04

## Discussion

**The magnitude of the outbreak:** the 2018 - 2020 measles outbreak in DR Congo was the largest ever measles outbreak documented. It affected multiple provinces and continued over a period of more than 2 years, despite multiple localized vaccination interventions and a nationwide response vaccination conducted between October 2019 - March 2020 [27].

The overall reported measles cases and deaths may have been underestimated due to some barriers in care seeking, especially with mild febrile rash cases or in very remote areas where medical care may not be sought at all, or when parents take sick children to traditional healers instead of health facilities, or as a result of gaps in surveillance and notification, among others. In addition, the suspected measles cases reported were mainly from health facilities, and data from active case search and line-listed cases from outbreak investigation were not systematically captured in all cases, with the likelihood of underestimation of the overall case count.

The analysis of the two surveillance datasets indicates a big difference between what was captured in the aggregate surveillance reporting system as compared to the case-based surveillance system. The fact that the country was experiencing a large outbreak means that surveillance officers in the field are not expected to continue taking samples but rather to document cases using line lists and confirm cases by epidemiological linkage. However, it is expected that line listed data with some basic epidemiological variables is captured in the case-based surveillance database. In the case of DR Congo, only 40,479 cases (8.8% of the 458,156 total suspected cases reported in the aggregate reporting system) were reported through the case-based surveillance system, of which only 13,414 (2.9%) of the total suspected cases had blood specimens collected for laboratory confirmation. According to the laboratory confirmation reported in the cased-based surveillance database, rubella virus circulated widely in DR Congo, at the same time as the measles outbreak. The occurrence of rubella will continue to confound the interpretation of aggregate surveillance data of suspected measles cases, and calls for the strengthening of laboratory confirmation and efforts to improve the quality of measles case-based surveillance.

**The context and the epidemiological characteristics of the outbreak:** measles outbreaks occur when the number of susceptible persons in the population reaches a critical threshold usually taken as the equivalent of one birth cohort. The occurrence of this large outbreak in DR Congo can be explained on the basis of a combination of several predisposing factors. While administrative measles vaccine coverage for the first dose (MCV-1) was often reported to be high, the WHO-UNICEF coverage estimates revealed that the annual national MCV-1 coverage had never reached 80% in the last two decades [13].

DR Congo has been implementing periodic measles supplemental immunisation activities (SIAs) as one of the strategies to bridge the immunity gaps among young children. The country implemented these measles SIAs throughout the past years in a phased manner, with part of the country being scheduled to complete the SIAs within a particular calendar year. This was done in order to address the limitations in terms of logistics and operations. However, suboptimal quality campaigns and prolonged inter-campaign intervals have contributed to critical accumulation of susceptible young cohorts and subsequent measles outbreaks [14,22,23].

Our analysis has shown that some provinces were more affected in terms of case load and/or the case fatality. The analysis also showed that the highest cumulative incidence rate of confirmed measles is reported among children aged less than 5, followed by children from 5 - 9 years of age. Only 41% of the lab confirmed measles cases aged less than 5 were vaccinated. Gaps in routine vaccination coverage have been reported as a root cause of measles outbreaks in other African countries such as Madagascar, Kenya, Cameroon, and Ethiopia [28-31]. Madagascar experienced a large measles outbreak in 2018 -2019 with 112,693 cases and 748 deaths reported on the background of an accumulation of unvaccinated children over a period of many years, leading to a significant proportion of cases occurring within the older childhood, adolescent and young adult population [28]. The age pattern of measles in this protracted outbreak is similar to measles outbreaks that occurred in the DR Congo in 2010-2012, in Cameroon in 2011, and in Guji zone in Ethiopia in 2015 [23,32,33]. The average age for acquiring measles depends on biological and epidemiological factors, mainly population immunity and birth rate. In settings with low population immunity, high birth rates and high population density, measles mostly affects children including infants and pre-school children.

Measles case fatality ratio is usually 1 - 3% in developing countries. However, higher case-fatality ratios may occur due to a young age at infection, crowding, underlying immune deficiency disorders, vitamin A deficiency, and lack of access to medical care [2]. In the DR Congo measles outbreak of 2011 - 2012, the case fatality ratio was 1.8%, and our findings from the 2018 - 2020 outbreak give the same result [23]. Ethiopia and Madagascar reported measles case fatality ratios of less than 1% during outbreaks in recent years [28,33].

**The factors contributing to the outbreak:** broader health system issues should be considered in the case of this large and protracted outbreak in DR Congo. The country has a large surface area, with a lot of hard-to-reach areas where huge logistical assets are needed to bring vaccination services in many remote areas. Health service provision was also often challenged by insecurity and protracted humanitarian situations in some provinces [16-19]. In the current outbreak, higher number of deaths and case fatality ratios were reported in provinces affected by humanitarian crises such as Kasaï and Sankuru. In addition, the national health system was challenged by concomitant outbreaks of yellow fever and Ebola virus disease (EVD) as well as recurrent outbreaks with circulating Vaccine Derived Polioviruses (cVDPVs) [5]. The delays in the implementation of planned preventive measles mass vaccination campaigns scheduled for early 2019, as well as the concurrent evolution of the EVD outbreak could have contributed to the progressively widening geographical spread and the magnitude of the measles outbreak.

**Routine immunisation strengthening and introduction of a second dose of measles vaccine in DR Congo:** in order to strengthen the immunization system in the DR Congo, an emergency plan for routine immunisation strengthening (called Mashako plan) was launched in 9 provinces in 2019, focusing on five key areas to improve vaccination coverage. These areas included: i) the increase the number of immunization sessions by 20%; ii) the reduction of vaccines stockouts by 80% in local health centers; iii) regular monitoring and evaluation; iv) monthly inspection of vaccination activities in health zones by inspectors and; v) the coordination and funding [34]. As of January 2022, the implementation of the Mashako plan has led to a significant increase in the number of vaccination service delivery sites providing regular services; the availability of vaccines has reached 79% as compared to 53% in 2019; 94% of vaccine refrigerators are functional; and the proportion of fully vaccinated children has increased by an average of 27% points in the 9 provinces. The ministry of health has now scaled up the Mashako plan to 23 provinces [35].

Like other African countries, the DR Congo was working towards the goal for measles elimination by 2020 [9]. In 2017, WHO issued a revised guidance on measles second dose introduction in all national immunization programs recommending that countries aiming at measles elimination should achieve at least 95% coverage with two doses equitably to all children in every district [1]. Measles second dose vaccination administered in the second year of life helps to reduce the accumulation of susceptible children by immunizing those who did not respond to the first dose. In addition, vaccination sessions in the second year of life provide an opportunity for infants who missed their vaccine doses to catch up with their required primary vaccine doses, thus increasing the overall numbers of immunized children [36]. However, the experience within the African Region is that introducing a second dose measles vaccination requires intensive preparations to generate demand, provide guidance and train health workers, as well as put in place the required logistics and monitoring systems beforehand [37].

**Limitations of the study:** the reporting of suspected measles cases is mostly done from the health facility level, and does not adequately capture mild cases which have not presented for treatment, or those cases occurring in remote areas. The measles case reporting in DR Congo, in the context of repeated measles outbreaks, has mostly relied on aggregate reporting of suspected cases, and it is a very small proportion of the cases which have benefited from lab testing and documentation through the case-based surveillance system. The aggregate surveillance reporting database does not provide granularity with regards to age, does not include critical information like vaccination status or laboratory confirmation. The line listing of cases and epidemiological linkage is not done regularly, and so were excluded from the analysis. The analysis of age-specific incidence was done using only the laboratory-confirmed measles cases. The case-based surveillance quality and performance indicators were not included in our analysis. The surveillance system is not designed to capture measles deaths in a comprehensive manner, since measles deaths can occur due to complications, weeks after the onset of the rash, and may not be accurately documented as such. In this study, we limited our analysis to the epidemiological characterization of the reported cases, and we did not factor in the various outbreak response interventions that were conducted in 2018 - 2020, which affected the evolution and the pattern of the epidemic.

## Conclusion

The DR Congo has been experiencing frequent measles outbreaks over the last decade. The outbreak that started in late 2018 was extensive and protracted, and it occurred in the face of persistently low routine immunisation coverage, delayed implementation of planned preventive SIAs, and multiple public health priorities in the country. The probable root cause of the outbreak is the wide gaps in the vaccination of eligible infants and young children. The country should invest resources to equitably raise routine vaccination coverage across the various parts of the country, and improve the timeliness and quality of SIAs. The Mashako plan is a promising approach to sustainably raise vaccination coverage, and the lessons and best practices should be documented and scaled up widely. The introduction of a second dose measles vaccine in the routine program should be implemented as a matter of priority, as it will provide the opportunity of building a second year of life platform for vaccination service delivery and improving coverage. This analysis has demonstrated the limitation in the granularity of data provided by the aggregate reporting system, which has very limited use for urgent decision making when it comes to disease control. Thus, there is a need to strengthen the case-based surveillance system along with the laboratory confirmation of suspected measles cases, as well as the detailed investigation of outbreaks and the capture of line-listed data in order to better understand the epidemiological trends and take appropriate decisions. In addition, urgent efforts are required to boost the preparedness to handle outbreaks of vaccine preventable diseases.

### What is known about this topic

- Measles is a highly contagious disease and occurs as periodic frequent outbreaks in countries with low routine immunisation coverage;
- DR Congo has not been able to attain 80% measles vaccine coverage according to the WHO -UNICEF coverage estimates, and has not introduced a second dose of measles vaccine in the national immunisation program;
- DR Congo has experienced large measles outbreaks mostly affecting young children in previous years.

### What this study adds

- DR Congo experienced wide-spread and protracted measles outbreaks in the face of persistently low routine immunisation coverage, delayed implementation of planned preventive SIAs, and multiple public health priorities;
- Failure to provide vaccination services to eligible children within the routine immunization program and mass campaigns has contributed to the large outbreak;
- Urgent efforts are required to improve routine vaccination and surveillance services, as well as the preparedness to handle outbreaks of vaccine preventable diseases.
